# Dental anomalies and their therapeutic implications: retrospective assessment of a frequent finding in patients with cleft lip and palate

**DOI:** 10.1186/s12903-022-02606-3

**Published:** 2022-12-01

**Authors:** A. K. Sander, E. Grau, A. K. Bartella, A. Kloss-Brandstätter, M. Neuhaus, R. Zimmerer, B. Lethaus

**Affiliations:** Department of Oral and Maxillofacial Surgery, University Hospital Leipzig, Liebigstr. 12, 04103 Leipzig, Germany

**Keywords:** Cleft lip/palate, CPO, Dental aplasia, Supernumerary teeth, Hypoplastic teeth, Orthognathic surgery, Orthodontic therapy

## Abstract

**Background:**

Orofacial clefts are characterized by a frequent occurrence of dental anomalies. Numerous studies demonstrate the high prevalence of dental aplasia, supernumerary teeth, and hypoplastic teeth in patients with cleft lip with/without cleft palate (CL/P), yet the therapeutic consequences are rarely discussed. This study explores prevalence, localization, and association between primary and secondary dentition in a large European collective and begins to evaluate the significance of dental anomalies in the therapeutic course of patients with CL/P.

**Methods:**

The medical reports of 1070 patients with different entities of CL/P who presented to our clinic within a 15-year investigation period were evaluated retrospectively. Dental anomalies were classified into three different diagnostic groups: dental aplasia, supernumerary teeth and hypoplastic teeth. The statistical analyses included studies of the frequency and localization of dental anomalies in different cleft entities as well as of the association between primary and secondary dentition and the therapeutic consequences.

**Results:**

Uni- or bilateral cleft lip and palate (CLP) (47.5%) occurred most frequently, followed by cleft palate only (CPO) (32.9%) and cleft lip with or without alveolus (CL ± A) (19.6%). Dental anomalies were found significantly more often on the side of the cleft. Aplastic permanent teeth were mostly found in patients with CLP (54.8%), while supernumerary permanent teeth occurred primarily in patients with CL ± A (21.7%). Patients with CPO presented dental aplasia but no patient with CPO showed supernumerary teeth. The occurrence of dental aplasia in the primary dentition significantly increases the probability of aplastic teeth in the permanent dentition. Dental anomalies, in particular dental aplasia, significantly increase patients’ need for subsequent orthodontic therapy and orthognathic surgery.

**Conclusion:**

Dental aplasia and hypoplasia are common in patients with CL/P not only in the cleft area but in the whole dentition. In the event of dental aplasia in the primary dentition, the frequency of aplastic teeth in the permanent dentition is significantly higher. Additionally, the need for therapeutic interventions, especially concerning orthognathic surgery, seems to be significantly higher in patients with CL/P who are affected by dental anomalies. Clinicians should take this into account when creating long-term treatment plans.

## Introduction

Orofacial clefts are among the most common anomalies in humans, the incidence in Europe is reported to be 1.36 in 1000 newborns [1]. The embryological development of the face and mouth is not only complex but also extremely sensitive to genetic and environmental influences. This results in a high occurrence of orofacial clefts, but also in a deficient understanding of their etiology [2].

The teeth form from the epithelium and underlying mesenchyme of the dental lamina and their morphogenesis is controlled by a highly complex interplay between specific signaling molecules, receptors and transcription factors [3]. There is a high occurrence of dental anomalies in patients with cleft lip and/or palate (CL/P) with studies reporting a prevalence of more than 60% [4] up to over 90% of patients showing any dental anomaly [5, 6]. Dental anomalies in the cleft region might be explained by the anatomical defect, deficiencies in mesenchymal tissue or the surgical interventions at an early age [7]. However, the generally increased number of dental anomalies, also on the contralateral side or in the mandibula, suggests there might be additional mechanisms involved, possibly in the form of shared genetic variations. For example, mutations in the transcription factor MSX1 are associated with orofacial clefting and non-syndromic hypodontia [8, 9].

The most prominent forms of dental anomalies are hypodontia, supernumerary, and hypoplastic teeth. The frequency of dental anomalies increases with cleft severity [10] though for supernumerary teeth, this could not be confirmed [11]. The findings are usually associated with the cleft side and occur especially often in the lateral incisor area of the maxilla [12].

This study aimed to evaluate the frequency and distribution of dental anomalies in a large collective of patients with cleft lip and/or palate and their possible impact on the patients’ treatment.

## Methods

The conduct of the study was approved by the ethical review committee, University Leipzig, Germany (IRB Board Number 00001750, 12–15-2020).

At our tertiary care medical center, an interdisciplinary consultation hour for patients with cleft lip and palate is held weekly. Parents or caregivers whose children underwent primary surgery at our clinic are recommended to visit annually after primary surgical reconstruction, in order to provide their children with a close follow-up for optimal care. In addition, the consultation is open to all patients with cleft lip and palate who are in need of further treatment or medical advice. This concerns mostly adult patients with the desire for aesthetic or functional corrective surgery but also children who have moved to the area after receiving the initial treatment at a different center for cleft lip and palate.

The present study evaluated the medical reports of all patients who were seen at our consultation hour between June 2005 and August 2020. This resulted in a total of 3470 examination reports pertaining to 1126 patients who were seen between one and eleven times during the examination period. The patient related parameters age, sex, cleft entity, and known syndromic disease were evaluated as well as information about medical findings and treatment recommendations. For this study, findings concerning the primary and secondary dentition including dental aplasia, hypoplasia and tooth gemination were of special interest. Dental hypoplasia was defined as teeth with changes in shape and hypomineralization.

The statistical analyses were performed using IBM SPSS (version 27; International Business Machines Corp., Armonk, NY, USA). The analyses included descriptive evaluation of the cohort as well as the Pearson’s chi-squared test for sets of unpaired categorical data to evaluate the probability of coincidental differences. In case of too small sample sizes, we used the Fisher’s exact test. The level of significance was accepted at *p* < 0.05.

To determine whether dental aplasia in the primary dentition increases the chances of aplastic teeth in the secondary dentition, we used Bayes’ theorem to compute conditional probabilities for patients with and without aplasia in the primary dentition. This analysis was performed using the software environment R (Version 4.1.1, R core team 2021). R was again used for graphical representation of data.

## Results

### Descriptive statistics and change of teeth

A total number of 3470 medical reports of 1126 patients was examined. After the exclusion of patients with incomplete reports, the statistical evaluation included data of 1070 patients. 57% (N = 610) of them were male and 43% (N = 460) were female. The average age of patients throughout all consultations was 10.15 years (range: 0 – 70). Comparing cleft entities, uni- or bilateral CLP (47.5%, N = 508) occurred most frequently, followed by cleft palate only (CPO) (32.9%, N = 352) and cleft lip with or without alveolus (CL ± A) (19.6%, N = 210). In 6.2% of patients (N = 66) the cleft appeared in the context of a syndromic disease or a Pierre Robin sequence.

Data referring to the primary dentition was available for 724 patients and data referring to the secondary dentition was available for 621 patients. Of 331 patients both, data of the primary and of the secondary dentition, could be evaluated. 56 patients were below the age of two at their last consultation, so no solid diagnosis could be made. The mean age when patients presented with the fully developed primary dentition was 3.56 years (SD: 1.18). The first phase of the mixed dentition was reached at a mean age of 6.58 (SD: 1.18) and the second phase at a mean age of 9.95 years (SD: 1.14). The mean age in which patients presented with complete permanent dentition was 13.48 years (SD: 1.52).

### Prevalence and distribution of dental anomalies in different cleft entities and sides

Out of 724 patients with complete data regarding the primary dentition, 11.1% presented one or more aplastic teeth. Concerning the secondary dentition, 621 patients were evaluated with 38.6% of patients showing dental aplasia. The mean number of aplastic permanent teeth was 0.76 (95% CI: 0.65 – 0.85; range: 0 – 13). Concerning the primary dentition, the mean number was 0.12 (95% CI: 0.09 – 0.15; range: 0 – 4). This means that the frequency is significantly higher in the permanent teeth and on average we find less than one aplastic tooth per patient in the permanent dentition.

**Fig. 1:**
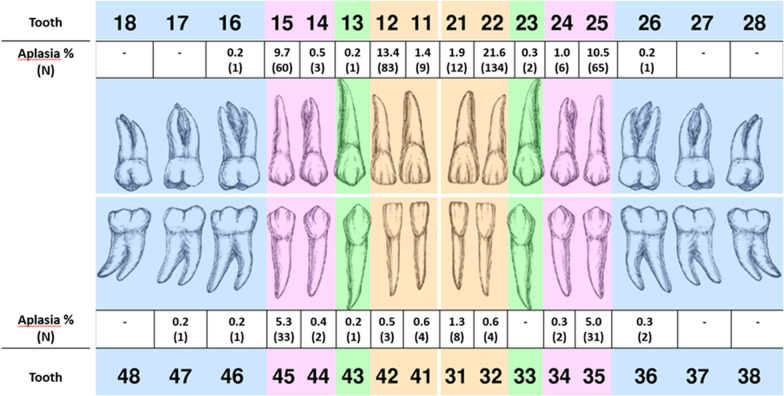
Prevalence of aplasia concerning single teeth in the secondary dentition (N = 621)

Figure 1 shows an odontogram presenting the distribution of dental aplasia within the secondary dentition.

**Fig. 2:**
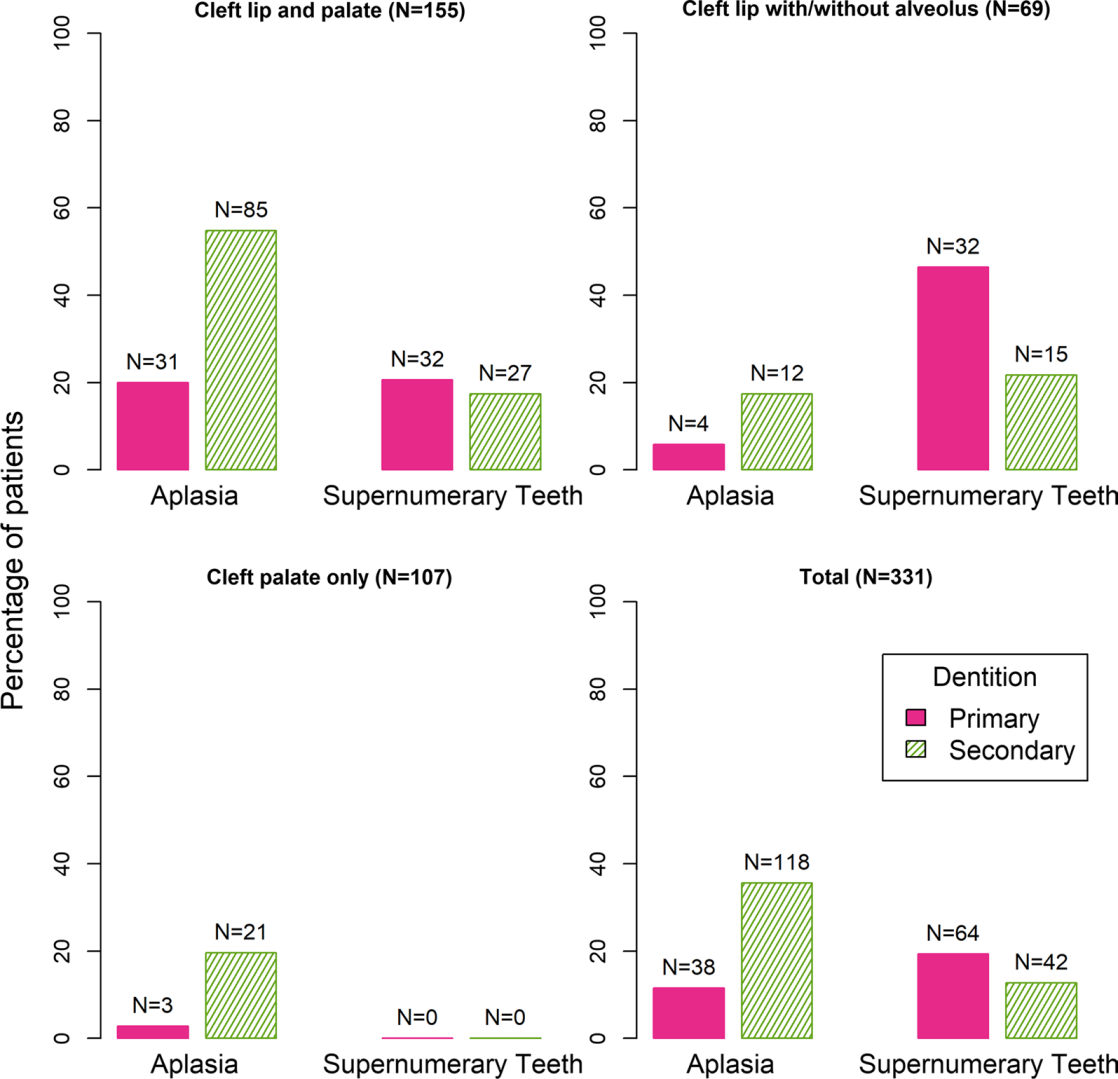
Prevalence of dental aplasia and supernumerary teeth in different cleft entities in the primary and secondary dentition

Figure 2 shows the frequency of dental aplasia and supernumerary teeth regarding the different cleft entities in the primary and secondary dentition. Aplasia in secondary dentition was most frequent in patients with CLP, followed by patients with CPO. Supernumerary teeth were more frequently found in the primary dentition concerning 19.3% (N = 64) of patients, compared to 12.7% (N = 42) of patients who showed supernumerary teeth in the permanent dentition.

To evaluate differences in cleft sides, patients were divided into four groups: (1) cleft entities concerning the right side and the (2) left side respectively, (3) bilateral clefts and (4) cleft palate only. Table 1 shows the frequency of cleft sides, the corresponding prevalence of dental aplasia, and the significance of associations between the two variables. Patients with bilateral clefts had the highest risk of dental aplasia in the permanent dentition (58.9%).

**Table 1:** Frequency of cleft sides, prevalences of dental aplasia and supernumerary teeth of the primary and secondary dentition and significance of associations between cleft side and aplasia/supernumerary teeth. N = 331, p < 0.05

Cleft side	Aplasia in primary dentition % (N)	*p*	Aplasia in secondary dentition % (N)	*p*	Supernumerary teeth in primary dentition % (N)	*p*	Supernumerary teeth in secondary dentition % (N)	*p*
Right (18.7%, N = 62)	14.5 (9)	> 0.05	32.3 (20)	> 0.05	19.4 (12)	> 0.05	17.7 (11)	> 0.05
Left (32.0%, N = 106)	16.0 (17)	> 0.05	41.5 (44)	> 0.05	30.2 (32)	< 0.05*	19.8 (21)	< 0.05*
Bilateral (16.9%, N = 56)	16.1 (9)	> 0.05	58.9 (33)	< 0.05*	35.7 (20)	< 0.05*	17.9 (10)	> 0.05
CPO (32.3%, N = 107)	2.8 (3)	< 0.05*	19.6 (21)	< 0.05*	0 (0)	< 0.05*	0 (0)	< 0.05*
Total (100%, N = 331)	11.5 (38)		35.6 (118)		19.3 (64)		12.7 (42)	

Significant numbers presented in italics and marked with an asterisk

### Association between primary and secondary dentition

The association between supernumerary teeth of the primary and of the secondary dentition was highly significant (*p* < 0.05). While hypoplastic teeth were found in 5.9% (N = 43) of patients in the primary dentition, the frequency in the secondary dentition was 17.7% (N = 110). There was no significant association between hypoplasia of teeth in the primary and secondary dentition.

Dental aplasia was distinctly more frequent in the permanent than in the primary dentition and most frequent in patients with CLP, affecting 54.8% of the entire group.

**Fig. 3:**
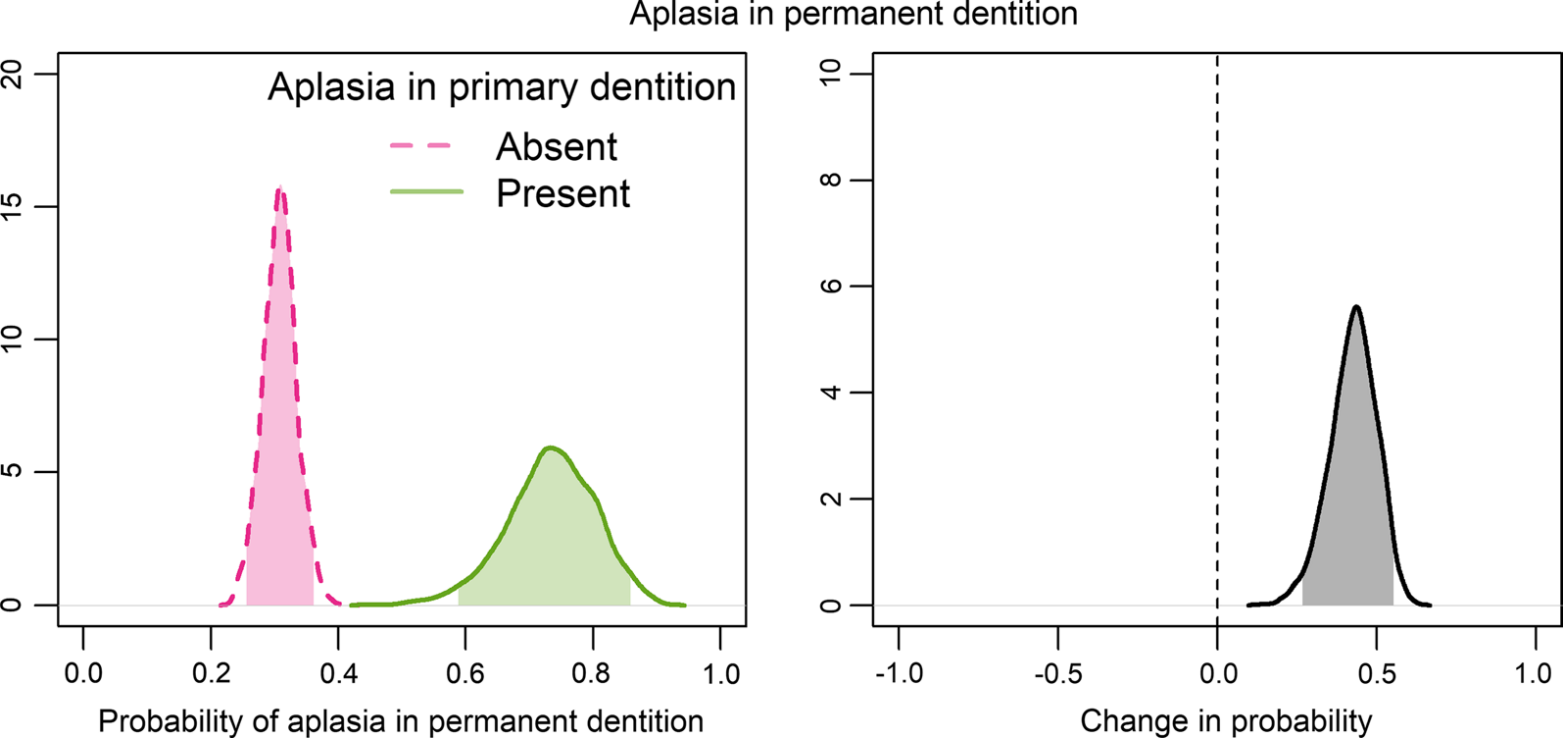
Probabilities of aplasia in permanent dentition for patients with and without aplasia in primary dentition (left) and change in probability of aplasia in the secondary dentition in case of aplastic teeth in the primary dentition (right)

Figure 3 shows conditional probabilities of aplasia in permanent dentition for patients with and without aplasia in primary dentition on the left and their respective difference on the right. Results show that the probability of aplasia in the secondary dentition is increased by on average 41.83% (95% CI 26.04 – 54.85%).

### Dental anomalies and their therapeutic implications

Table 2 shows the associations between dental aplasia and therapeutic decisions that were made during the follow-up period. We found significant associations between all three kinds of dental anomalies of the permanent dentition and orthodontic therapy (*p* < 0.05). This was especially obvious for patients affected by dental aplasia and supernumerary teeth: orthodontic therapy was recommended to 75.9% of patients with dental aplasia. Adversely, orthodontic therapy was recommended to only 36.7% of patients without this condition. Of patients affected by supernumerary teeth, 84.1% received orthodontic therapy.

**Table 2:** Associations between dental aplasia and therapeutic decisions using Chi-Square test and Fisher’s exact test (p < 0.05)

	Primary dentition (N = 724)	Secondary dentition (N = 621)
Orthodontic therapy	< 0.05*	< 0.05*
Alveolar bone grafting	< 0.05*	< 0.05*
Orthognathic surgery	> 0.05	< 0.05*
Logopedic therapy	> 0.05	> 0.05

Significant numbers marked with an asterisk

A significant association was also found between logopedic therapy and supernumerary teeth of the secondary dentition (*p* < 0.05).

Dental anomalies were additionally found to be highly associated with the therapeutic indication of orthognathic surgery. Of 24 patients who underwent orthognathic surgery, 16 patients showed dental aplasia, 5 showed hypoplasia, and 1 patient showed supernumerary teeth. The association between dental aplasia and orthognathic surgery was consequently found to be highly significant (*p* < 0.05).

## Discussion

There is an intuitive association between clefts including the alveolar ridge and dental agenesis or hypoplasia in that specific area. Many case–control studies evaluated the type and number of dental anomalies compared to the healthy population to show that patients with CL/P are more likely to be affected by several kinds of aberrations concerning the dental system. Two meta-analyses from 2012 and 2021 have addressed the issue and came to the same conclusion: evidence suggests that a higher number of dental anomalies is noted in individuals born with orofacial clefts [18, 19].

Various explanations are used for this correlation. On the one hand, the failed fusion of the maxillary and medial nasal prominences might lead to mesenchyme insufficient to develop into a healthy tooth [7]. The anatomic defect of the cleft might lead to insufficient calcification of the lateral incisor in the cleft area. Another factor to be considered is the early surgical interventions for reconstruction of lip and palate, that might impair the development and calcification of the anterior teeth crowns and induce teeth displacement and rotation in the posterior region [7, 20, 21]. A study by Korolenkova et al. assessed the role of external etiological factors in the development of dental anomalies and found a significant relation between their prevalence and the treatment protocol. Early orthodontic therapy to reposition the maxillary segments and reduce soft tissue tension, palatal defect after primary palatoplasty and primary periosteoplasty each had a significant effect on the prevalence of dental aplasia, hypoplasia and frontal teeth enamel defects [22]. The evaluation of prevalences of dental anomalies might therefore also allow the comparison with other collectives to rule out excessive external influences in one clinic.

On the other hand, the higher incidence of dental aplasia outside the cleft region points in the direction of underlying genetic causes. Evidence suggests that some genetic variations might induce both orofacial clefting and hypodontia, supposedly by affecting the regulation of ectodermal-mesenchymal signaling pathways [23–25]. Ultimately it is most likely an intricate combination of internal and external factors resulting in a high prevalence of various dental anomalies in patients with CL/P. Nonetheless, many of the studies evaluating the correlation between orofacial clefts and dental anomalies excluded cleft variations that did not affect the alveolar ridge (cleft lip only, cleft palate only) [19]. Our data showed for patients with CPO, that the prevalence of aplasia in secondary dentition was higher than in the healthy European population [26], but no patient showed supernumerary teeth. Supernumerary teeth in patients with CL + A or CLP occurred in the cleft area only. This might indicate, that while dental aplasia in patients with CL/P is at least partly caused by genetic variations, supernumerary teeth develop mainly due to the physical interruption of the alveolar ridge.

Dental aplasia of the permanent dentition was seen most often in the maxillary lateral incisors, but was also found in maxillary and mandibular premolars, maxillary medial incisors and very rarely in any other tooth (Fig. 1). In our collective the change of teeth showed no noticeable deviation from the norm, which contradicted one of our working hypotheses.

While there is a large body of literature on the causes of dental agenesis and aplasia, the therapeutic implications of dental anomalies in the treatment of patients with CL/P have been discussed rarely. Maxillary hypoplasia is a common finding in patients with CL/P and to this day, there is no consensus about its reasons. Postsurgical scarring of the palate has been found to contribute to the maxillary growth inhibition whereas the effect of lip surgery seems to be of smaller impact [13, 14], and has on the contrary been discussed to induce maxillary growth [15]. Missing lateral incisors clearly have been found to play a major role in the development of the maxilla [16, 17]. Our data shows a higher need for orthodontic treatment and also for orthognathic surgery compared to cleft patients without dental aplasia. The high prevalence of dental anomalies (> 90%) in patients in need of orthognathic surgery confirms the theory that dental agenesis correlates with maxillary hypoplasia and might even predict the need for Le Fort I advancement in cleft patients [17].

In any case one must consider that dental anomalies might correlate with the cleft width and severity, thus the higher treatment need might be partly caused by a more complex initial situation [26, 27].

For parents and caregivers of patients with orofacial clefts the question of anomalies to be expected in the permanent dentition usually arises at an early age. We could show that the occurrence of aplasia in the primary dentition in cleft patients significantly increases the probability of dental aplasia in the permanent dentition. In general, we found a higher frequency of dental aplasia in the permanent dentition compared to the primary dentition, which is in accordance with the literature [28]. This applied to all cleft entities (Table 1). The opposite relationship was observed for supernumerary teeth: while no case was observed in patients with CPO, patients with CLP or CL ± A showed a significantly higher prevalence of supernumerary teeth in the primary compared to the permanent dentition.

Patients with a left-sided or bilateral cleft of any manifestation presented with a significantly higher risk for aplasia of permanent dentition and supernumerary teeth of primary dentition than patients with other cleft entities. This is in contrast to Möller et al. whose results suggest that right-sided clefts of patients with CLP are more susceptible to supernumerary teeth [26].

In summary, the generally high frequency of dental anomalies differs significantly between patients with different cleft entities and also between primary and secondary dentition. While dental aplasia occurs mainly in the cleft area, it appears-with a reduced frequency-in the whole maxillary and mandibulary dentition. Supernumerary teeth on the other hand were documented in the cleft region only, suggesting that while orofacial clefts and dental aplasia share some underlying genetic mechanisms, the connection between orofacial clefts and supernumerary teeth is mainly mechanical. Dental anomalies, especially dental aplasia, significantly increase patients’ need for orthodontic therapy and orthognathic surgery. Physicians involved in the treatment of patients with orofacial clefts need to be aware of the high frequency of dental anomalies and the therapeutic implications in this patient population.

## Data Availability

The datasets analyzed within the current study are available on Open Science Framework, https://osf.io/mu74s/, https://doi.org/10.17605/OSF.IO/MU74S.

## Abbreviations

CI: Confidence interval
CL ± A: Cleft lip with/without alveolar involvement
CLP: Cleft lip and palate
CL/P: Cleft lip and/or palate
CPO: Cleft palate only
SD: Standard deviation
